# Interplay Between Pollution and Avian Influenza Virus in Shorebirds and Waterfowl

**DOI:** 10.1007/s10393-025-01707-z

**Published:** 2025-03-07

**Authors:** Tobias A. Ross, Junjie Zhang, Michelle Wille, Alexandros G. Asimakopoulos, Veerle L. B. Jaspers, Marcel Klaassen

**Affiliations:** 1 Centre for Integrative Ecology, School of Life and Environmental Sciences, Deakin University, 75 Pigdons Road, Geelong, VIC 3216 Australia; 2 Department of Chemistry, Norwegian University of Science and Technology (NTNU), 7491 Trondheim, Norway; 3 Sydney School for Infectious Diseases, School of Life and Environmental Sciences and School of Medical Sciences, The University of Sydney, Sydney, NSW Australia; 4 Department of Microbiology and Immunology, Peter Doherty Institute for Infection and Immunity, The University of Melbourne, Melbourne, VIC Australia; 5 WHO Collaborating Centre for Reference and Research on Influenza, Peter Doherty Institute for Infection and Immunity, Melbourne, VIC Australia; 6 Department of Biology, Norwegian University of Science and Technology (NTNU), 7491 Trondheim, Norway; 7 Victorian Wader Study Group, Thornbury, VIC 3071 Australia

**Keywords:** Avian influenza, shorebird, duck, pFASs, immunomodulation, wildlife health

## Abstract

Anthropogenic pollution may disrupt wildlife immune function and increase susceptibility to, and ability to withstand, infection. Of particular concern is avian influenza virus (AIV), which in its low-pathogenic form is endemic in many wild bird populations, notably waterfowl and shorebirds, and in its high-pathogenic form poses a threat to wildlife, livestock and people. Many pollutants have immunomodulative properties, yet little is known about how these pollutants affect AIV infection risk specifically. We examined concentrations of known immunomodulatory compounds, per- and polyfluoroalkyl substances (PFASs), and assessed their influence on AIV infection in three reservoir species, red-necked stint (*Calidris ruficollis,* n = 121), pacific black duck (*Anas superciliosa,* n = 57) and grey teal (*Anas gracilis*, n = 62). Using data on viral prevalence (cloacal/oropharyngeal swabs) and seroprevalence (AIV anti-nucleoprotein antibodies), we found no significant effect of PFASs pollution (total PFASs < 0.01–470 ng/g in red-necked stint, < 0.01–600 ng/g in pacific black duck and 0.3–200 ng/g in grey teal) on infection status in our three species. This may be due to relatively low pollutant concentrations, but we cannot rule out possible population culling through a synergy of pollution and infection stressors. We therefore recommend further studies on infection incidence in more polluted populations or species.

**Supplementary Information:**

The online version contains supplementary material available at 10.1007/s10393-025-01707-z.

## Introduction

Pollution threatens wildlife species all around the globe through a diversity of effects. At high enough concentrations, chemical pollution can be acutely toxic, causing direct mortality (Katagi & Fujisawa, 2021). At sub-lethal levels, pollutants may cause physiological harm both individually (Dietz et al., 2019), and when interacting with one another. These interactions can result in other additive or even synergistic harmful effects (Dennis et al., 2020). Physiological effects of pollutants are diverse and may include disruption of both endocrine systems (Dietz et al., 2019; Sebastiano et al., 2021) and reproductive function (Dietz et al., 2019), an increase in oxidative stress (Valavanidis et al., 2006) and immunomodulation (Castaño-Ortiz et al., 2019; Vallverdú-Coll et al., 2019). Immunomodulation can change the way organisms interact with pathogens in their environment, thus impacting individuals’ susceptibility to and recovery from disease with knock-on effects on the epidemiology of pathogens.

Interactions between pollution and pathogens can be complex. Direct effects have been studied in a range of pollutants; lead, for example, increases susceptibility to infections by altering T-lymphocyte response in domestic chickens (*Gallus gallus*) (Vallverdú-Coll et al., 2019). The same effects have also been reported from air contaminants from oil sands (including ozone, sulphur dioxide and polycyclic aromatic hydrocarbons) in wild tree swallows (*Tachycineta bicolor*) (Cruz-Martinez et al., 2015). Mercury pollution has been shown to cause a reduction in natural antibodies in barnacle goslings (*Branta leucopsis*) (Han, van den Berg, Loonen, Mateo, & van den Brink, 2022). Expression of different immune genes in embryonic chicken fibroblasts can be reduced by both polychlorinated biphenyls (PCBs: Badry et al., 2020) and per-/polyfluoroalkyl substances (PFASs: Castaño-Ortiz et al., 2019). Indirect interactions with immune function can also emerge with pollution potentially reducing an animal’s body condition resulting in limited availability of resources to mount an immune response to combat infection (Teitelbaum et al., 2022). While immunomodulatory effects of many pollutants have been well studied in vitro and in captive birds, studies of both pollution and disease simultaneously in wild birds have rarely been conducted (Teitelbaum et al., 2022).

PFASs are a growing immunomodulatory contaminant group that consist of partially or fully fluorinated carbon chains. This structure lends them both hydro- and lipophobic properties and high stability (Buck et al., 2011). These properties have led to the use of PFASs in many products, including firefighting foams, textiles and kitchen utensils. PFASs are rapidly increasing in diversity with some estimates suggesting over 14,000 are currently in circulation (US Environmental Protection Agency, 2022) with little regulation in place (UNEP, 2022). PFASs have become highly prevalent in wildlife due to their occurrence in the environment, especially in aquatic ecosystems (Xiao, 2017). These habitats are where species such as Anseriformes (waterfowl) and Charadriiformes (shorebirds) frequently forage, resulting in exposure via the consumption of contaminated water and prey (De Silva et al., 2021).

The immunomodulative properties of PFASs can affect both adaptive and innate immunity of wildlife. In birds, Peden-Adams et al. (2008) showed a reduction of the adaptive response in response to the phytohemagglutinin (PHA) test in chickens (*Gallus gallus domesticus*) exposed to perfluorooctane sulfonic acid (PFOS) while in the egg, whereas Smits and Nain (2013) showed a significant reduction of the T cell-dependent antibody response in Japanese quails (*Coturnix japonica*). Effects on innate immunity in chickens include a downregulation in the expression of immune genes nuclear factor kappa-light-chain-enhancer of activated B cells (*NF-κB),* interleukins 8 (*IL-8*) and 4 (*IL-4*), all of which are involved in the inflammation response (Castaño-Ortiz et al., 2019). Further complicating matters is the tendency of PFASs to persist in tissues such as blood, where perfluorinated compounds such as PFOS have been shown to have half-lives of 230 days in birds (Tarazona et al., 2015), which may exacerbate the effects of these pollutants on wildlife.

Migratory birds of the East-Asian Australasian flyway (EAAF) spending the non-breeding period in Australia may experience exposure to low-pathogenic avian influenza virus (AIV), but are potentially also exposed to highly pathogenic avian influenza while in Asia (Wille et al., 2019). Highly pathogenic avian influenza causes severe morbidity and mortality in wild birds and is currently causing a panzootic with profound impacts on birds, globally (Wille & Barr, 2022). The virus is most readily transmitted by faecal material and is thus easily spread in species that forage in shallow water and in large numbers (Wille et al., 2023). Active infections of AIV can last from 1–2 weeks (Latorre-Margalef et al., 2014), while antibodies may remain in the birds’ blood for up to a year (Bethany J Hoye et al., 2011). AIV is an endemic virus in Australian wild birds including (but not limited to) waterfowl and shorebirds (Wille et al., 2023) and can particularly occur in aquatic environments (Pathak et al., 2018). It is in these same environments that may also increase the birds’ exposure to PFASs, as discussed above. Furthermore, AIV is often associated with other co-infections (Wille et al., 2018) and may thus function as an indicator of overall susceptibility to infection.

In this study, we investigated the effect of PFAS pollution on the infection risk of AIV in three of the pathogen’s prime Australian host species. We focussed on three avian species: one species of shorebird, red-necked stint (*Calidris ruficollis*, order Charadriiformes) and two waterfowl species (order Anseriformes): pacific black duck (*Anas superciliosa*) and grey teal (*Anas gracilis*). Long-term AIV prevalence and AIV seroprevalence levels in these three species were established at 3% and 10%, 7% and 50% and 6% and 55%, respectively, which are among the highest AIV prevalence values measured in birds in Australia (Wille et al., 2023). Furthermore, serological evidence of highly pathogenic avian influenza infection has previously been found in red-necked stint (Wille et al., 2019). All three species can inhabit similar habitats in Australia and thus may have similar exposure to both PFASs and AIV in the same habitat. However, red-necked stints also migrate via an extremely polluted region (including PFASs), China’s Yellow Sea (Muir & Miaz, 2021; Xiao et al., 2017), thereby increasing their chances of exposure to both pollution and disease, highlighting the potential risk of interplay between both PFASs and AIV. As PFASs (and PFOS in particular) have been shown to downregulate the innate immune response (Castaño-Ortiz et al., 2019), we expected that with increasing exposure to these pollutants the prevalence of both AIV and AIV antibodies would increase.

## Methods

### Ethics Statement

All birds were captured and sampled under approval of Deakin University Animal Ethics Committee (permit numbers A113-2010, B37-2013, B43-2016, B39-2019), Wildlife Ethics Committee of South Australia (2011/1, 2012/35, 2013/11) and Philip Island Nature Park Animal Ethics Committee (SPFL20082).

### Sample Collection

Red-necked stints were captured on the coast of Victoria, at the Western Treatment Plant (WTP) and in Western Port Bay, both near Melbourne, Australia. Pacific black ducks were sampled at the WTP, and on farmland near Geelong, also in Victoria. Grey teal were also sampled at the WTP and Innamincka Regional Reserve in South Australia. Catches of birds occurred from 2011 to 2020. Red-necked stint were captured using cannon-nets in collaboration with the Victorian Wader Study Group (VWSG), as part of ongoing shorebird banding work starting 1978 (Minton, 2006). Ducks were caught using baited walk-in traps and mist nets. All birds were blood sampled, and oropharyngeal and cloacal swabs were taken. Of each bird, cloacal and oropharyngeal swab samples were collected using sterile swabs and immediately stored in virus transport medium (VTM, brain heart infusion [BHI] broth-based medium [Oxoid] with 0.3 mg/ml penicillin, 5 mg/ml streptomycin, 0.1 mg/ml gentamicin and 2.5 g/ml, amphotericin B), either separately (pre-March 2014) or together (March 2014 onwards). Swab samples in VTM were kept refrigerated for up to a week, before being stored at below − 80 °C. Blood samples (typically 200 µl) were collected from the brachial vein of each bird using Sarstedt Microvette capillary tubes. Samples were refrigerated and left to clot, next centrifuged within 6–24 h after collection to separate red blood cells (RBC) from serum, after which they were stored at below − 20°C. Serum was used to test for the presence of AIV antibodies, while RBC was used for PFAS analyses.

Across the entire influenza surveillance study, we collected 1512 sample sets from red-necked stints, 446 from pacific black ducks and 660 from grey teals. None of these birds were positive for an active infection of highly pathogenic AIV; this has yet to be detected in wild birds in Australia (Grillo et al., 2015; Wille et al., 2022, 2023). We selected the samples of all 41 AIV-positive red-necked stints, 29 AIV-positive pacific black ducks and 29 AIV-positive grey teals. These samples were matched with the samples from 68, 28 and 33 AIV-negative birds, respectively. The AIV-negative sample sets were stratified-randomly selected, ensuring at least 10 AIV-negative individuals were included from each of the sites where AIV-positive birds were sampled. Serum samples from the same birds were also used for AIV antibody analyses with the exception of two pacific black ducks and three grey teals for which we failed to collect serum. For red-necked stint, we included an additional 12 serum samples for serological analysis. Where samples had been found to be seropositive in prior studies that also use this data, only some were analysed for their specific strain of AIV, where it was found that less than 2% of these seropositive samples were positive for highly pathogenic AIV antibodies (Wille et al., 2019). Based on this, we assumed that our positive and seropositive samples were all low-pathogenic strains. Overall, total numbers of individuals involved across all samples were 121 red-necked stints, 57 pacific black ducks and 62 grey teals.

### AIV Screening

Samples were screened for AIV following established methods, described in Wille et al. (2023). Briefly, RNA was extracted from swab samples, which were assayed for AIV using quantitative reverse transcriptase real time PCR targeting a short fragment of the matrix gene (Spackman et al., 2002). A cycle threshold (Ct) cut-off of 40 was used. In cases where oropharyngeal and cloacal swabs were collected separately (pre-March 2014), results were combined and a bird was deemed AIV-positive if either swab returned a positive result. Blood serum samples were assayed for anti-nucleoprotein antibodies of AIV using the Multi Screen Avian Influenza Virus Antibody Test Kit (IDEXX, Hoppendorf, The Netherlands) following manufacturer’s instructions.

### Chemical Analyses of Blood Samples

All RBC samples were analysed for 12 different PFASs (full details provided in Table S1), which were broadly grouped as carboxylates (PFPA, PFHxA, PFOA, PFNA, PFDA, PFUnA, PFDoA, PFTrA, PFTeA) and sulfonates (PFBS, PFOS). We also calculated a total PFAS group, consisting of all carboxylates and sulfonates listed above, as well as PFOSA. Analyses were conducted using methodology adapted from Trimmel et al. (2021). In brief, ~ 50 mg of red blood cell samples was weighted in a 1.5 mL polypropylene tube and 10 µL of PFOA-^13^C_8_ and PFOS-^13^C_8_ (1000 µg/L) was added. After adding 0.3 mL methanol containing 1% ammonium formate, the sample was vortexed for 30 s and ultrasonicated for 30 min. Then, the sample was centrifuged for 5 min at 3500 rpm. Finally, the supernatant was transferred into a 1.5 mL injection vial with an insert vial after purification by Hybrid-SPE. The sample extract was stored at -20 °C until analysis by UPLC-MS/MS.

### Statistical Analysis

All statistical analyses were conducted using R Version 4.2.0, in RStudio 2022.02.0 Build 443. All PFASs were grouped into carboxylates (ΣPFCAs), sulfonates (ΣPFSA) and total PFASs (ΣPFAS) as outlined above. All concentrations within each category were summed. All non-detected values of individual compounds were replaced with 0.015 ng/g, equivalent to half the minimum quantification limit of detection for all compounds. When summed categories were calculated, any totals where no compounds were detected were also set at 0.015 ng/g. For each of the three focal species, we first ran two-sample t tests on the grouped PFAS data (ΣPFCAs, ΣPFSAs and ΣPFASs) from each region, to determine if there were pollution differences between regions.

We used generalised linear mixed effects models in R package lme4 (Bates et al., 2015) (function glmer, family binomial) to investigate AIV status (positive or negative) in each species, as a function of each of ΣPFCAs, ΣPFSAs and ΣPFASs, while including region as a random intercept (i.e. 3 × 3 = 9 models). We also ran this same structure of models across all samples, including species as a second random intercept alongside region (i.e. 3 models). Finally, we ran the same series of models (both by species, and all species combined with species and region as random intercepts), in which ΣPFCAs and ΣPFSA concentrations were both entered simultaneously as explanatory variables (i.e. 3 species-specific, and 1 all samples combined = 4 models). Overall, we thus ran a total of 16 models for AIV prevalence. To account for multiple comparisons, we adjusted the significance thresholds using the Benjamini–Hochberg correction within each of the three groups of models (i.e. we adjusted the significance threshold for the group of nine species-specific models, again for the group of three combined-species models in which species was included as a random effect, and finally for the three species-specific models that included both ΣPFCAs and ΣPFSAs as explanatory variables). We ran the same set of models using AIV serostatus (positive or negative) instead of AIV status as dependent variable, culminating in a total of 32 models testing the effect of PFASs on AIV status and serostatus. The same structuring of models was run when including log-transformed rather than untransformed (raw) pollutant concentrations, to minimise the effect of particularly high concentrations of PFASs on our model results. Thus, across both raw and log-transformed pollutant data, we ran a grand total of 64 models.

## Results

**Figure 1:**
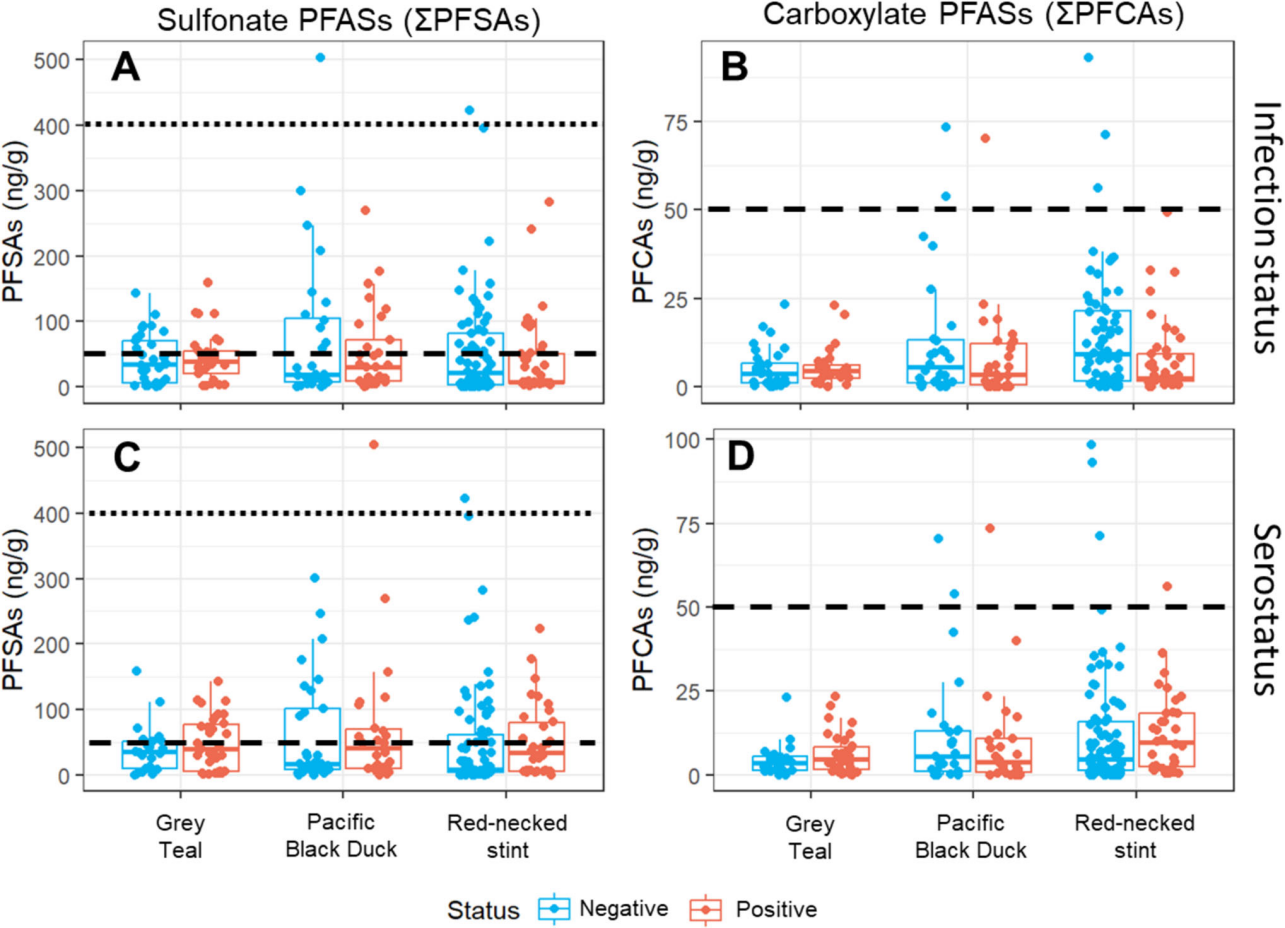
Concentrations (ng/g) of summed sulfonate (A,C) PFASs (ΣPFSAs) and summed carboxylate (B, D) PFASs (ΣPFCAs) in red blood cells from grey teal (*Anas gracilis*, n = 62), pacific black duck (*Anas superciliosa*, n = 57) and red-necked stint (*Calidris ruficollis*, n = 121). **A** and **B** show infection status, while **C** and **D** show serostatus. Birds that were positive are represented in red, and negative in blue. Dennis et al. (2021)’s proposed lowest observable adverse effects level (LOAEL) for PFOS in adult bird livers (400 ng/g) is shown by the dotted line in A and C, while the LOAEL in juvenile bird livers (50 ng/g) is shown by the dashed line in all four plots.

Of the 12 individual pollutants that we targeted, only PFOS, PFDoA and PFUnA were detected in more than 50% of our samples (94%, 72% and 60%, respectively, see Tables S2 and S3). When summed into overall categories, ΣPFCAs, ΣPFSAs and ΣPFASs were detected in 90%, 95% and 98% of all samples, respectively, regardless of species (Tables 1 and 2). In all but 7 samples (3%), ΣPFCAs concentrations were below 50 ng/g which was the lowest observable adverse effects level (LOAEL) in juvenile avian liver tissue proposed by (Dennis et al., 2021). These samples were from four red-necked stints (56, 71, 93 and 98 ng/g) and three pacific black ducks (54, 70 and 74 ng/g), comprising the exception. ΣPFSAs by contrast were higher in concentration, with 82 samples (34%) exceeding the 50 ng/g threshold (Fig. 1), comprising of 40 red-necked stints (50.2–423 ng/g) and 21 pacific black ducks (52–504 ng/g) and 21 grey teal (50.9–205 ng/g). We found that all ΣPFCAs, ΣPFSAs and ΣPFASs were significantly higher at the WTP in all species (*p* < 0.05, see Table S4 for summary concentrations), with one exception where no significant differences between regions were found for ΣPFCAs in pacific black duck. A complete range of detection rates and overall concentrations of PFAS groupings by AIV status and AIV serostatus are shown in Table 1 and Table 2, respectively.

**Table 1:** Median and range concentrations of grouped PFASs (in ng/g) in red blood cells of red-necked stint (Calidris ruficollis), pacific black duck (Anas superciliosa) and grey teal (Anas gracilis), based on the AIV infection status of each individual.

Comp	All samples			Red-necked stint					
		Raw	Log	Raw	Log	AIV-negative n = 68		AIV-positive n = 41	
	%	*P*_*all*_	*P*_*all*_	*P*_*stint*_	*P*_*stint*_	Med	Range	Med	Range
ΣPFSAs	94.6	0.215	0.152	0.304	**0.038**	21.4	(< 0.01–423)	7.17	(1.26–282)
ΣPFCAs	90	**0.021**	0.318	**0.041**	0.782	9.40	(< 0.01–93.2)	2.36	(< 0.01–49.2)
ΣPFASs	97.5	0.186	0.306	0.221	0.188	38.3	(< 0.01–467)	10.5	(1.84–331)

Comp	Pacific black duck						Grey teal					
	Raw	Log	AIV-negative n = 28		AIV-positive n = 29		Raw	Log	AIV-negative n = 33		AIV-positive n = 29	
	*P*_*duck*_	*P*_*duck*_	Med	Range	Med	Range	*P*_*teal*_	*P*_*teal*_	Med	Range	Med	Range
ΣPFSAs	0.341	0.843	19	(< 0.01–504)	30.1	(< 0.01–270)	0.932	0.423	34.3	(< 0.01–143)	38.5	(0.81–159)
ΣPFCAs	0.311	0.239	5.45	(< 0.01–73.5)	3.41	(< 0.01–70.4)	0.963	0.413	3.68	(< 0.01–23.3)	4.56	(< 0.01–23)
ΣPFASs	0.353	0.598	27.9	(< 0.01–599)	36.6	(1.98–292)	0.847	0.366	50.3	(0.3–174)	54.2	(1.7–205)

Where < 0.01 is noted, this means the concentration was below the limit of quantification. Emboldened and bold *p* values indicate statistical significance between AIV-negative and AIV-positive individuals, for models containing raw or log-transformed PFAS concentrations. Where a value is recorded as NA, this compound was not detected in the species. When *p* values were adjusted using a Benjamini–Hochberg correction, no significant effects of pollution on AIV infection status were found.

**Table 2:** Median and range concentrations of grouped PFASs in red blood cells (in ng/g) of red-necked stint (Calidris ruficollis), pacific black duck (Anas superciliosa) and grey teal (Anas gracilis), based on AIV serostatus of each individual.

Comp	All samples			Red-necked stint					
		Raw	Log	Raw	Log	AIV-seronegative n = 87		AIV-seropositive n = 34	
	%	*P*_*all*_	*P*_*all*_	*P*_*stint*_	*P*_*stint*_	Med	Range	Med	Range
ΣPFSAs	94.6	0.600	0.066	0.870	0.116	12.0	(< 0.01–423)	18.7	(< 0.01–282)
ΣPFCAs	90	0.845	0.272	0.994	0.115	8.0	(< 0.01–93.1)	4.69	(< 0.01–98.2)
ΣPFASs	97.5	0.609	0.132	0.887	0.125	24.0	(< 0.01–467)	22.3	(< 1.32–343)

Comp	Pacific black duck						Grey teal					
	Raw	Log	AIV-seronegative n = 29		AIV-seropositive n = 26		Raw	Log	AIV-seronegative n = 24		AIV-seropositive n = 35	
	*P*_*duck*_	*P*_*duck*_	Med	Range	Med	Range	*P*_*teal*_	*P*_*teal*_	Med	Range	Med	Range
ΣPFSAs	0.801	0.561	17.5	(< 0.01–300)	40.7	(< 0.01–504)	0.374	0.503	34.9	(< 0.01–159)	40.2	(0.81–143)
ΣPFCAs	0.645	0.683	5.46	(< 0.01–70.4)	3.78	(< 0.01–73.5)	0.270	0.390	3.66	(< 0.01–23)	4.56	(< 0.01–23.3)
ΣPFASs	0.815	0.837	25.4	(< 0.01–373)	45.5	(< 0.01–599)	0.364	0.603	50.6	(0.3–205)	55.4	(1.2–174)

Where < 0.01 is noted, this means the concentration was below the limit of quantification. Emboldened and shaded *p* values indicate statistical significance between seropositive and seronegative individuals,, for models containing raw or log-transformed PFAS concentrations. Where a value is recorded as NA, this compound was not detected in the species. When *p* values were adjusted using a Benjamini–Hochberg correction, no significant effects of pollution on AIV serostatus were found.

The persistence of PFASs in the blood and long detection time of AIV antibodies allowed us to investigate the presence of any relationships between PFASs and both active infection as well as past infection. Across all 64 models (both raw and log-transformed pollutant concentrations), no significant effects were found for the influence of PFAS pollution on AIV prevalence and seroprevalence, except for seven models (*p* values for models with raw and log-transformed pollutant concentrations for each species, and overall sample population provided in Tables 1 and 2). Using raw data, significant effects were found for ΣPFCAs on AIV infection status for all species combined, and for ΣPFCAs on AIV infection status for red-necked stints when modelled individually. In models with log-transformed PFAS data, neither of these effects were observed. On the other hand, a significant effect of log-transformed ΣPFSAs was found for red-necked stints. In both raw and log-transformed models, ΣPFCAs were also found to have a significant effect on AIV infection status of red-necked stints when ΣPFSAs were also included in the same model. Finally, when both ΣPFCAs and ΣPFSAs were modelled together for all species combined, ΣPFCAs had a significant effect in models using both raw and log-transformed data, while ΣPFSAs had a significant effect only in the model using log-transformed concentrations for both pollutant groups. No significant effect of pollution on serostatus was found in any model.

In all models in which ΣPFCAs were found to have a significant effect, ΣPFCAs were found to be lower in birds infected with AIV: across all species modelled together (AIV-positive median: 3.44 ng/g, range: < 0.01–98.2 ng/g; AIV-negative median: 5.25 ng/g, range: < 0.01–93.2 ng/g; slope_raw_ = -0.028, *p*_*raw*_ = 0.021), when modelled simultaneously with ΣPFSAs (slope_raw_ = -0.036, *p*_*raw*_ = 0.038; slope_log_ = -0.596, *p*_*log*_ = 0.009), and in red-necked stints when ΣPFCAs were modelled individually (AIV-positive median: 2.36 ng/g, range: < 0.01–49.2 ng/g; AIV-negative median: 9.40 ng/g, range: < 0.01–93.2 ng/g; slope_raw_ = -0.037, *p*_*raw*_ = 0.041) and with ΣPFSAs (slope_raw_ = -0.048,* p*_*raw*_ = 0.049; slope_log_ = -0.048 *p*_*log*_ = 0.049). Where significant effects were found for ΣPFSAs, these indicated higher pollutant concentrations in birds with AIV infections: when modelled alone via log transformation in red-necked stints alone (slope_log_ = 0.912, *p*_*log*_ = 0.038), and in all species when modelled concurrently with log-transformed ΣPFCAs (slope_log_ 0.644 = , *p*_*log*_ = 0.006)*.* However, these significant effects may be spurious due to the number of models we tested. Applying a Benjamini–Hochberg correction for multiple comparisons suggested no significance for all models tested, aside from the two models of AIV prevalence across all species that included both ΣPFCAs and ΣPFSAs as explanatory variables and showed a negative relationship between ΣPFCAs and AIV prevalence in both raw and log-transformed models, and a positive relationship between ΣPFSAs and AIV prevalence in the log-transformed model only.

## Discussion

Our results, encompassing concentrations of 12 per-/polyfluorinated compounds, and both AIV infection and serostatus in three different species did not suggest that infection of AIV in these birds is influenced by their PFAS burdens. Of the 64 models run, only seven significant correlations were detected with *p* values just below 0.05, which could well be spurious given the number of models that were run, as was indicated by our Benjamini–Hochberg corrected *p* values. Our general finding of no (or few) significant correlations is in contrast to a previous in vitro study where PFASs were demonstrated to have immunomodulatory effects in birds (Castaño-Ortiz et al., 2019). If such effects were present in the current study, we would have expected actively AIV-infected or previously AIV-infected birds to have higher concentrations of PFASs in their system.

We expect that the absence of a correlation between PFAS pollution and AIV in this study was because our observed PFAS concentrations were too low to elicit immunomodulatory effects. In the in vitro study by Castaño-Ortiz et al. (2019), exposure to PFOS was at 22 mg/L, likely far higher than any exposure our study birds experienced in the environment. Indeed, even though PFAS pollution often varied between sample sites, the concentrations we observed were low relative to ‘predicted no-effects concentrations’ in serum presented by Newsted et al. (2005), the lowest of which was 150 ng/g. Total PFAS concentrations in all but three individuals’ (two red-necked stints and one pacific black duck) were also low compared to LOAELs of 400 ng/g in adult bird liver tissues proposed by Dennis et al. (2021). These LOAELs were calculated based on decreased adult weight gain. Unfortunately, the disparity between sample matrices (liver vs. red blood cells) means we cannot draw direct comparisons between our study and Dennis et al. (2021)’s proposed toxicity thresholds. Nevertheless, concentrations of PFASs in liver tend to be higher than those in blood (Gebbink & Letcher, 2012), and as such, we can consider Dennis et al. (2021)’s liver LOAELs to be conservative estimates of toxicity in blood. The true number of our samples that exceed the adult LOAEL from Dennis et al. (2021) is likely to be higher. Aside from the three individual exceptions mentioned above, the low concentrations we observed may be considered remarkable for birds that inhabit putatively polluted wastewater treatment plants (all focal species) or migrate via highly polluted environments (red-necked stint migrating via e.g. the Yellow Sea) (Muir & Miaz, 2021). Nevertheless, in 105 individuals, the total PFASs concentrations found were higher than the lowest LOAELs posited by Dennis et al. (2021) (50 ng/g in liver for young birds, based on decreased successful hatching), which suggested there may be some risk to juvenile birds in our population. The absence of specific data on immunomodulation thresholds means we cannot definitively state whether our concentrations are below levels to cause effect. However, our results did not yet suggest that this is the case.

It is worth noting that some individuals had particularly elevated PFASs concentrations (e.g. one pacific black duck was observed with 599 ng/g of PFOS, with the next highest concentration observed in a red-necked stint at 467 ng/g, see Fig. 1), while being negative for both AIV infection. However, no conclusions can be based on such rare extreme cases due to of a) the very small sample size of individuals with elevated PFASs and b) the level of exposure of these pacific black ducks and red-necked stints to AIV (10% seroprevalence: Wille et al., 2023) increasing stochasticity in the occurrence of infection. It is clear though that individuals can accrue a high PFAS load without necessarily incurring infections.

An alternative explanation for the low PFASs concentrations, and minimal significant effects (aside from seven out of the 64 models suggesting a negative relationship between AIV infection and pollution burden), is that the absence of an effect is due to population culling. Population culling is the phenomenon whereby sick, or in this case, polluted individuals do not survive and are thereby ‘culled’ from the population, such that we never or infrequently detect them when sampling. In our sample population, there was clearly the possibility for individuals to accrue elevated PFAS burdens, as demonstrated by the 2 birds with PFSAs concentrations greater than 400 ng/g (see Fig. 1). In the absence of additional stressors such as disease, birds may be able to withstand elevated concentrations. Congruently, wild birds have limited documented signs of disease associated with low-pathogenic avian influenza beyond a reduction in daily movement in mallards (*Anas platyrhynchos*) (Van Dijk et al., 2015) and poorer performance in foraging or migrating Bewick’s swans (*Cygnus columbianus*) (Hoye et al., 2016; Van Gils et al., 2007). The limited extent of these signs is such that infection alone has little to no direct effect on the survival of its host (Maxted et al., 2012). However, both the negative effect of pollution in isolation and the potential combined effect of pollution and disease might even cause death, such that individuals with downregulated immune responses due to pollution may be unable to survive the combined effects of PFASs and the virus (Guruge et al., 2009). Such individuals may therefore have been lost to our study. Indeed, in the majority of models where we found significant effects of pollution on AIV infection status, birds with lower pollution were more likely to be AIV positive. The significant model results could indicate that infected birds with higher concentrations are unable to survive the added pressures of both and therefore ‘culled’ from the population. Hence, it is possible that we did not sample highly polluted birds that also had avian influenza infections, because they did not survive. This is particularly pertinent for migrating shorebirds which also deal with the additional stressors of physiological effects of migration including immunomodulation (Buehler et al., 2010). Indeed, there is some evidence of migratory culling, whereby migration may remove sick individuals (McKay & Hoye, 2016). This effect could be more pronounced under pollution stress, which could become more toxic due to the thermal stress from exertion during migration (Gordon, Johnstone, & Aydin, 2014). This is a possible explanation as to why the only significant single-species models found were models involving data from red-necked stints, as these are the only migratory species included in the study.

Two other factors that we did not account for in our models may have influenced the infection status and contamination of our studied birds: age and sex. With regard to age, contaminants such as PFASs are reputed to be bioaccumulative (Houde et al., 2011), which suggests that older birds may accrue higher concentrations of PFASs than their younger conspecifics. Indeed, this has been previously seen in PFCASs in adult versus immature red-necked stints sampled at the WTP (Ross et al., 2023). Similarly, older birds have had greater time to be exposed to AIV and thus may be more likely be AIV seropositive; conversely, juvenile birds that have not previously been exposed (and thus have no antibodies to the virus) may be more likely to have an active infection (Wille et al., 2023). There is also some evidence that sex may play a role, where females may have lower concentrations of pollution as they can sequester pollution loads in their eggs (Newsted et al., 2005). However, neither sex nor age were consistently identifiable for all of our sampled birds, and thus, we did not include these variables in our models.

Overall, our birds were all sampled in environments with relatively low PFASs concentrations, with the most polluted being processed wastewater at the WTP. In other regions of the world, PFAS pollution may be higher (Muir & Miaz, 2021), as is the prevalence of AIV and therefore the likelihood of a birds’ exposure to the virus (Wille & Barr, 2022). In these regions, the effects of pollution-mediated immunomodulation may be much stronger. The consequences of widespread immunomodulation could be severe, for populations of wild birds, and for human health and poultry industries. Our findings of no clear evidence of PFAS-mediated immunomodulation in Australia cannot discount effects in more heavily polluted regions of the world where exposure to more dangerous high pathogenicity AIV strains is more prevalent. The current global circumstances of increased highly pathogenic avian influenza transmission amongst wild birds in the northern hemisphere highlight the necessity to continue monitoring potential drivers of host susceptibility such as pollution, so that we may garner a better understanding of the threats the interactions of these stressors pose to wild birds.

## Supplementary Information

Below is the link to the electronic supplementary material.

Supplementary file1 (DOCX 249 KB)
