# Supplementary material for: Interplay Between Pollution and Avian Influenza Virus in Shorebirds and Waterfowl
Source: Ecohealth. 2025 Mar 7;22(2):222–32. doi: 10.1007/s10393-025-01707-z (PMC12259721; doi:10.1007/s10393-025-01707-z)
Supplement: Supplementary file 1 — Supplementary file1 (DOCX 249 KB) [file 10393_2025_1707_MOESM1_ESM.docx]

**SUPPLEMENTAL MATERIALS: Interplay between pollution and avian influenza virus infection in waterbirds**

**Table S1** Chemical names and formulae of PFASs

| **Compound Abbreviation** | **Full name** | **Structure** | **CAS** | **Formula** |
| --- | --- | --- | --- | --- |
| PFPA | Perfluoropentanoic acid |  | 2706-90-3 | C_5_HF_9_O_2_ |
| PFHxA | Perfluorohexanoic acid | 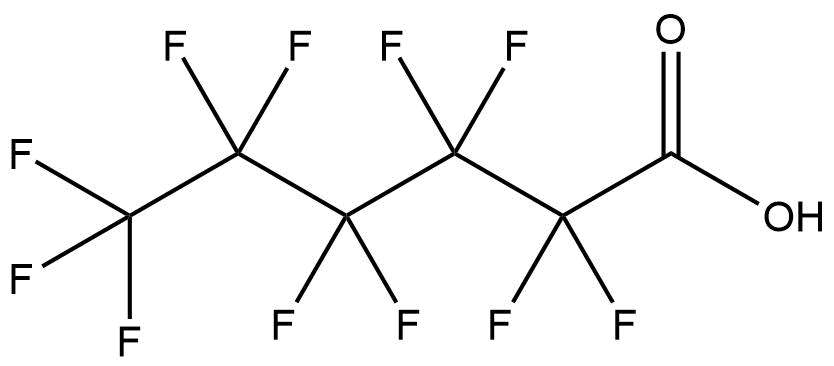 | 307-24-4 | C_6_HF_11_O_2_ |
| PFOA | Perfluorooctanoic acid | 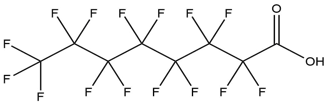 | 335-67-1 | C_8_HF_15_O_2_ |
| PFNA | Perfluorononanoic acid | 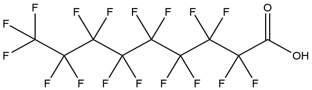 | 375-95-1 | C_9_HF_17_O_2_ |
| PFDA | Perfluorodecanoic acid | 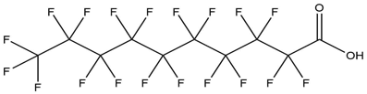 | 335-76-2 | C_10_HF_19_O_2_ |
| PFUnA | Perfluoro-undecanoic acid | 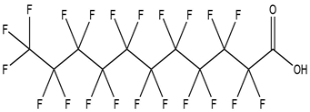 | 2058-94-8 | C_11_HF_21_O_2_ |
| PFDoA | Perfluoro-dodecanoic acid | 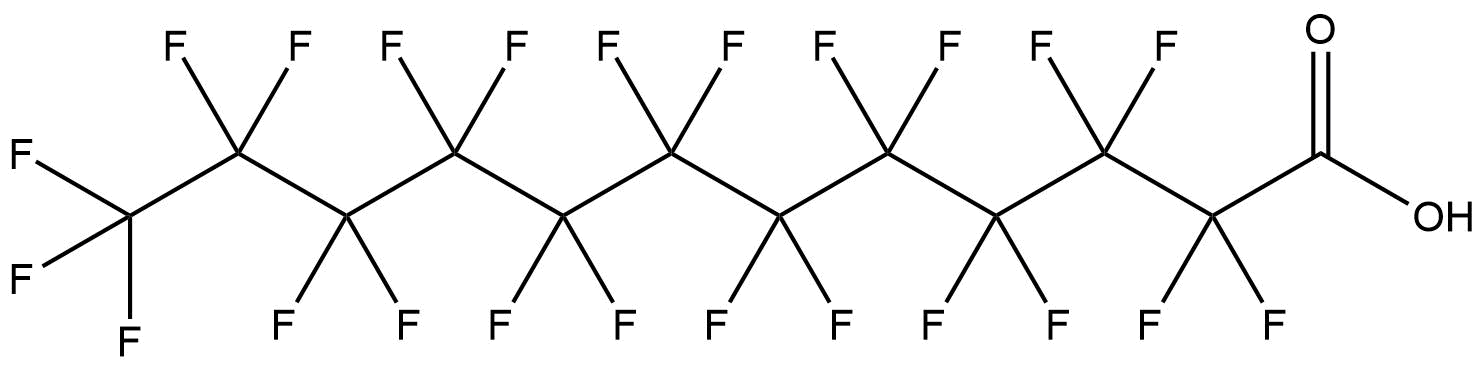 | 307-55-1 | C_12_HF_23_O_2_ |
| PFTrA | Perfluoro-tridecanoic acid | 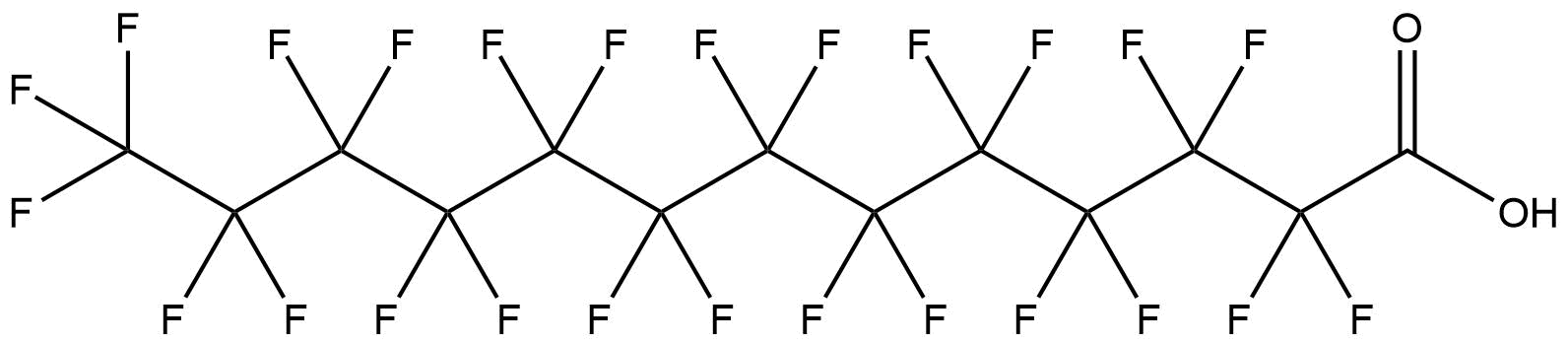 | 72629-94-8 | C_13_HF_25_O_2_ |
| PFTeA | Perfluoro-tetradecanoic acid | 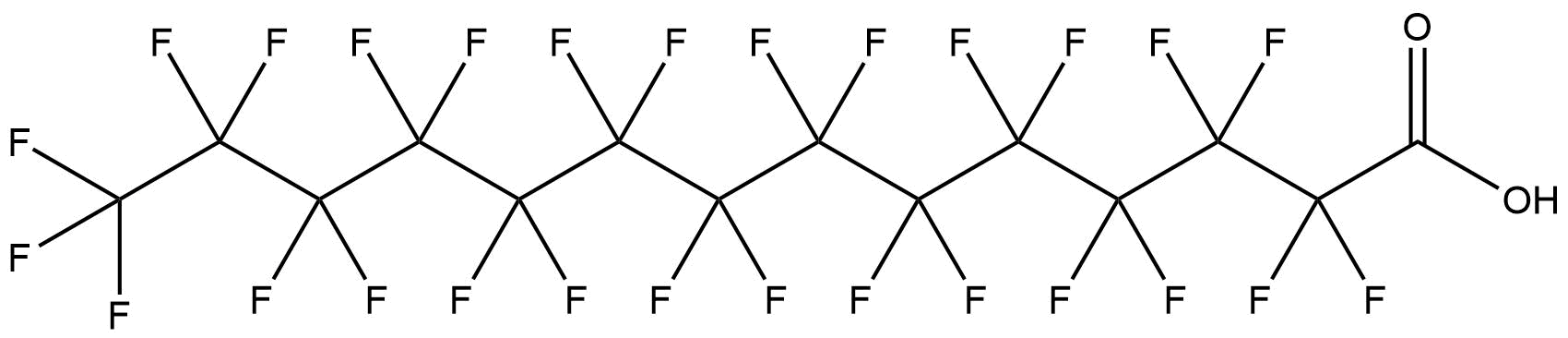 | 376-06-7 | C_14_HF_27_O_2_ |
| PFBS | Perfluorobutane-sulfonic acid |  | 375-73-5 | C_4_HF_9_SO_3_ |
| PFOS | Perfluorooctano-sulfonic acid |  | 1763-23-1 | C_8_HF_17_O_3_S |
| PFOSA | Perfluorooctane sulfonamide | 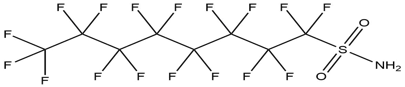 | 754-91-6 | C_8_H_2_F_17_NO_2_S |

**Table S2:** Summary concentrations (median, range) of all target PFASs with regard to the AIV infection status of each species, as well as overall detection frequencies of each compound for all samples.

| Compounds |  | Red-necked stint | | | | Pacific Black Duck | | | | Grey Teal | | | |
| --- | --- | --- | --- | --- | --- | --- | --- | --- | --- | --- | --- | --- | --- |
|  |  | AIV-negative  n=68 | | AIV-positive  n=41 | | AIV-negative  n=28 | | AIV-positive  n=29 | | AIV-negative  n=33 | | AIV-positive  n=29 | |
|  | Detection % | Median | Range | Median | Range | Median | Range | Median | Range | Median | Range | Median | Range |
| PFOS | 94.6 | 20.6 | (<0.01-396) | 7.17 | (1.26-282) | 19 | (<0.01-504) | 30.1 | (<0.01-270) | 34.3 | (<0.01-143) | 38.5 | (0.81-159) |
| PFBS | 2.1 | <0.01 | (<0.01-0.737) | <0.01 | (<0.01-5.39) | <0.01 | (<0.01-<0.01) | <0.01 | (<0.01-1.48) | <0.01 | (<0.01-<0.01) | <0.01 | (<0.01-<0.01) |
| PFPA | 1.25 | <0.01 | (<0.01-24.0) | NA | NA | <0.01 | (<0.01-<0.01) | <0.01 | (<0.01-<0.01) | <0.01 | (<0.01-<0.01) | <0.01 | (<0.01-<0.01) |
| PFHxA | 1.3 | <0.01 | (<0.01-7.6) | <0.01 | (<0.01-0.67) | <0.01 | (<0.01-<0.01) | <0.01 | (<0.01-<0.01) | <0.01 | (<0.01-<0.01) | <0.01 | (<0.01-<0.01) |
| PFOA | 31.7 | <0.01 | (<0.01-12.8) | 0.89 | (<0.01-8.17) | <0.01 | (<0.01-1.49) | <0.01 | (<0.01-1.35) | <0.01 | (<0.01-2.04) | 0.41 | (<0.01-2.36) |
| PFNA | 41.7 | <0.01 | (<0.01-15.5) | <0.01 | (<0.01-8.49) | 0.52 | (<0.01-3.36) | <0.01 | (<0.01-4.27) | 0.48 | (<0.01-3.05) | 0.54 | (<0.01-1.54) |
| PFDA | 37.9 | 0.66 | (<0.01-19.8) | <0.01 | (<0.01-9.14) | <0.01 | (<0.01-6.49) | <0.01 | (<0.01-13.7) | <0.01 | (<0.01-1.31) | <0.01 | (<0.01-2.81) |
| PFUnA | 60.4 | <0.01 | (<0.01-18.5) | 0.37 | (<0.01-6.79) | 1.39 | (<0.01-25.8) | 0.74 | (<0.01-9.79) | 0.91 | (<0.01-6.32) | 1.84 | (<0.01-9.65) |
| PFDoA | 72.5 | 0.515 | (<0.01-16.2) | 0.77 | (<0.01-13.8) | 1.29 | (<0.01-20.5) | 1.06 | (<0.01-40.7) | 0.93 | (<0.01-6.85) | 1.15 | (<0.01-6.33) |
| PFTrA | 42.9 | <0.01 | (<0.01-28.3) | <0.01 | (<0.01-13.6) | 0.278 | (<0.01-17.4 | <0.01 | (<0.01-5.51) | 0.52 | (<0.01-3.73) | <0.01 | (<0.01-2.71) |
| PFTeA | 39.6 | 0.192 | (<0.01-23.4) | <0.01 | (<0.01-10.9) | <0.01 | (<0.01-10.4 | <0.01 | (<0.01-12.7) | <0.01 | (<0.01-2.93) | <0.01 | (<0.01-2.96) |
| PFOSA | 50 | <0.01 | (<0.01-26.1) | <0.01 | (<0.01-14.0) | 0.56 | (<0.01-17.9) | 1.39 | (<0.01-36.5) | 4.86 | (<0.01-26.9) | 5.83 | (<0.01-21.6 |

**Table S3:** Summary concentrations (median, range) of all target PFASs with regard to the AIV serostatus of each species, as well as overall detection frequencies of each compound for all samples.

| Compounds |  | Red-necked stint | | | | Pacific black duck | | | | Grey teal | | | |
| --- | --- | --- | --- | --- | --- | --- | --- | --- | --- | --- | --- | --- | --- |
|  |  | AIV-seronegative  n=87 | | AIV-seropositive  n=34 | | AIV-seronegative  n=29 | | AIV-seropositive  n=26 | | AIV-seronegative  n=24 | | AIV-seropositive  n=35 | |
|  | Detection % | Median | Range | Median | Range | Median | Range | Median | Range | Median | Range | Median | Range |
| PFOS | 94.6 | 12.0 | (<0.01-396) | 17.9 | (<0.01-282) | 17.5 | (<0.01-300) | 40.7 | (<0.01-504) | 34.9 | (<0.01-159) | 40.2 | (0.81-143) |
| PFBS | 2.1 | <0.01 | (<0.01-0.73) | <0.01 | (<0.01-5.39) | <0.01 | (<0.01-1.48) | <0.01 | (<0.01-<0.01) | <0.01 | (<0.01-<0.01) | <0.01 | (<0.01-<0.01) |
| PFPA | 1.25 | <0.01 | (<0.01-24) | <0.01 | (<0.01-8.07) | <0.01 | (<0.01-<0.01) | <0.01 | (<0.01-<0.01) | <0.01 | (<0.01-<0.01) | <0.01 | (<0.01-<0.01) |
| PFHxA | 1.3 | <0.01 | (<0.01-7.6) | <0.01 | (<0.01-7.67) | <0.01 | (<0.01-<0.01) | <0.01 | (<0.01-<0.01) | <0.01 | (<0.01-<0.01) | <0.01 | (<0.01-<0.01) |
| PFOA | 31.7 | <0.01 | (<0.01-11.1) | <0.01 | (<0.01-12.8) | <0.01 | (<0.01-1.49) | <0.01 | (<0.01-0.44) | <0.01 | (<0.01-0.93) | <0.01 | (<0.01-2.36) |
| PFNA | 41.7 | <0.01 | (<0.01-15.5) | <0.01 | (<0.01-27.6) | 0.54 | (<0.01-4.27) | 0.092 | (<0.01-3.36) | 0.5 | (<0.01-1.54) | 0.62 | (<0.01-3.05) |
| PFDA | 37.9 | 0.700 | (<0.01-19.8) | <0.01 | (<0.01-10) | 0.46 | (<0.01-13.7) | <0.01 | (<0.01-6.49) | <0.01 | (<0.01-2.81) | <0.01 | (<0.01-1.9) |
| PFUnA | 60.4 | <0.01 | (<0.01-18.5 | <0.01 | (<0.01-20.5) | 1.59 | (<0.01-19.5) | 1.37 | (<0.01-25.8) | 0.92 | (<0.01-9.65) | 1.88 | (<0.01-8.49) |
| PFDoA | 72.5 | 0.510 | (<0.01-16.2) | 0.56 | (<0.01-13.8) | 1.53 | (<0.01-40.7) | 1.26 | (<0.01-20.5) | 0.815 | (<0.01-6.33) | 1.08 | (<0.01-6.85) |
| PFTrA | 42.9 | <0.01 | (<0.01-28.3) | <0.01 | (<0.01-25.5) | 0.54 | (<0.01-9.79) | <0.01 | (<0.01-17.4) | 0.505 | (<0.01-2.71) | 0.47 | (<0.01-3.73) |
| PFTeA | 39.6 | <0.01 | (<0.01-16.7) | 1.21 | (<0.01-23.4) | <0.01 | (<0.01-12.7) | <0.01 | (<0.01-10.4) | <0.01 | (<0.01-2.04) | <0.01 | (<0.01-2.96) |
| PFOSA | 50 | <0.01 | (<0.01-26.1) | <0.01 | (<0.01-14.0) | 0.47 | (<0.01-15) | 1.31 | (<0.01-36.5) | 5.31 | (<0.01-21.6) | 5.47 | (<0.01-26.9) |

**Table S4:** Summed PFAS median and range concentrations for each species at each sample site. Where concentrations are recorded as ‘<0.01’, these were below the limit of quantification.

| Site | Compound | Species | | | | | | | | |
| --- | --- | --- | --- | --- | --- | --- | --- | --- | --- | --- |
|  |  | Red-necked Stint | | | Pacific Black Duck | | | Grey Teal | | |
|  |  | n | Median (ng/g) | Range (ng/g) | n | Median (ng/g) | Range (ng/g) | n | Median (ng/g) | Range (ng/g) |
| Western Treatment Plant (WTP), Victoria | ΣPFCAs | 62 | 15.8 | <0.01-98.2 | 33 | 9.12 | <0.01-73.51 | 47 | 4.85 | 0.61-23.3 |
|  | ΣPFSAs |  | 62.8 | <0.01-423 |  | 71.46 | <0.01-504 |  | 44.82 | 4.87-158.85 |
|  | ΣPFASs |  | 92.4 | 467 |  | 101 | <0.01-599 |  | 56.62 | 5.85-205 |
| Western Port Bay (WPB), Victoria | ΣPFCAs | 60 | 1.63 | <0.01-24.0 | -- | -- | -- | -- | -- | -- |
|  | ΣPFSAs |  | 4.53 | <0.01-49.0 |  | -- | -- |  | -- | -- |
|  | ΣPFASs |  | 6.37 | <0.01-57.3 |  |  |  |  |  |  |
| Geelong, Victoria | ΣPFCAs | -- | -- | -- | 24 | 1.21 | <0.01-70.37 | -- | -- | -- |
|  | ΣPFSAs |  | -- | -- |  | 10.54 | <0.01-58.89 |  | -- | -- |
|  |  |  |  |  |  | 16.17 | <0.01-70.37 |  |  |  |
| Innamincka, South Australia | ΣPFCAs | -- | -- | -- | -- | -- | -- | 15 | 0.64 | <0.01-7.12 |
|  | ΣPFSAs |  | -- | -- |  | -- | -- |  | 2.31 | <0.01-7.64 |
|  | ΣPFASs |  |  |  |  |  |  |  | 2.83 | 0.3-9.6 |

'--' is used to denote the absence of data as the species was not sampled at this site.
